# Identification of Key Gene Related to Matrisome in HBV-Associated Liver Cirrhosis via Bioinformatics Analysis

**DOI:** 10.1155/ijh/5532643

**Published:** 2025-08-25

**Authors:** Ram Prasad Chaulagain, Yelona Shrestha, Khuzin Dinislam, Shizhu Jin

**Affiliations:** ^1^Department of Gastroenterology and Hepatology, Second Affiliated Hospital of Harbin Medical University, Harbin, China; ^2^National Academy of Medical Science, Bir Hospital, Kathmandu, Nepal; ^3^Department of General Chemistry, Bashkir State Medical University, Ufa, Republic of Bashkortostan, Russia

**Keywords:** ECM genes, extracellular matrix, hepatocellular carcinoma, liver cirrhosis, matrisome

## Abstract

**Background:** Hepatitis B virus (HBV)–associated liver cirrhosis, characterized by progressive fibrosis and regenerative nodule formation, remains a critical public health concern due to its high risk of progression to hepatocellular carcinoma (HCC). The matrisome—comprising extracellular matrix (ECM) components such as collagens, laminins, fibronectin, glycoproteins, and proteoglycans—plays a pivotal role in disease pathogenesis. Previous studies have shown that HBV infection modulates ECM composition and activates fibrogenic responses through hepatic stellate cells, contributing to cirrhosis and eventual HCC development. However, key ECM-related genes driving HBV-induced cirrhosis remain poorly understood.

**Methods:** Bulk RNA-seq data from 30 normal and 30 HBV-related cirrhotic liver tissues were analyzed. Differentially expressed genes (DEGs) were identified using the Limma package based on thresholds of *p* < 0.01 and |log2 fold change| > 1. ECM-related genes were curated from the Molecular Signatures Database (MsigDB). Functional significance was assessed via random forest classification (*accuracy*: 91%, *recall*: 90%), Gene Ontology (GO), and Kyoto Encyclopedia of Genes and Genomes (KEGG) pathway enrichment analyses.

**Results:** Among 14,470 analyzed genes, 2125 were upregulated and 3689 downregulated in cirrhotic tissues. Upregulated genes (*COX6B1*, *RPS10*) were linked to metabolic reprogramming, while downregulated genes (*PLCG2*, *ARHGEF12*) implicated immune dysregulation. A subset of 274 ECM-related DEGs (178 upregulated, 96 downregulated) was identified, including *CTSA*, *CRELD2*, *MAPK10*, and *ITGA1*. Pathway analysis highlighted dysregulation of Ras/MAPK and ERBB signaling pathways associated with fibrogenesis and tumorigenesis.

**Conclusions:** This bioinformatics study delineates key matrisome–associated genes and pathways in HBV-related cirrhosis, offering novel insights into potential biomarkers and therapeutic targets. Further validation in larger cohorts is warranted to confirm clinical relevance.

## 1. Introduction

Progressive fibrosis and regenerative nodular development are hallmarks of liver cirrhosis, a serious public health concern. Viral hepatitis, alcohol, nonalcoholic fatty liver disease, and autoimmune diseases are the leading causes of cirrhosis worldwide, and liver disease accounts for 4% of all deaths worldwide [1]. The World Health Organization stated that the Hepatitis B virus (HBV) caused 820,000 deaths and infected over 1.5 million new cases in 2019, affecting approximately 296 million people worldwide. The Polaris Observatory group's 2022 modeling research calculated that the worldwide HBV prevalence was 3.2%, or approximately 257.5 million cases [2]. In China, the seroprevalence of hepatitis B surface antigen (HBsAg) ranges from 3.7% to 10.4%, with a total of 74.6 million chronic HBV patients, making it the country with the largest number of chronic HBV cases worldwide [3]. Among Chinese patients with HBV-related cirrhosis, approximately one-third (31.5%) developed HCC over five years, with a cumulative incidence of 22.9% (95% CI: 20.8–25.2%) [4]. Furthermore, studies estimate the annual incidence of hepatocellular carcinoma (HCC) in cirrhotic patients to be 2%–5% [5]. Similarly, 30% of individuals with chronic HBV infection develop liver cirrhosis and HCC because of persistent liver inflammation and fibrosis [6]. The lack of screening facilities and low public awareness have been attributed to the increase in HBV-associated cirrhosis [7]. Although there are a number of methods to detect cirrhosis early, including blood tests, liver enzymes, and imaging tools, biopsy remains the most reliable procedure [8]. Computed tomography (CT), magnetic resonance imaging (MRI), and ultrasound are noninvasive imaging techniques that are expensive and inaccessible in developing countries. Serum biomarkers and hepatic stiffness measurements are becoming useful in detecting asymptomatic early-stage cirrhosis [9]. MicroRNAs (miRNAs) and other circulating nucleic acid biomarkers have shown promise as diagnostic, prognostic, and therapeutic tools for liver diseases, particularly HBV-HCC [10]. Therefore, serum markers offer a noninvasive method for the early screening and diagnosis of liver cirrhosis.

The term “matrisome” refers to the extracellular matrix (ECM) and its constituent proteins, including collagen, laminins, fibronectin, glycoproteins, and proteoglycans [11]. Maintaining tissue homeostasis and regulating cellular processes such as adhesion, migration, and signaling depend on the matrisome [12]. The matrisome maintains an ideal microenvironment and controls cellular activity to maintain tissue homeostasis. The ECM influences cell survival, proliferation, and differentiation by interacting with cells via cell adhesion receptors such as integrins. To maintain a balance between cell development and apoptosis, it provides biochemical and mechanical cues that guide cell behavior and reactions to external stimuli [13]. The matrisome also helps regulate waste clearance, nutrition exchange, and blood flow, all of which are essential for maintaining liver homeostasis [14]. Numerous cytokines, signaling molecules, and growth factors are stored in the matrisome. To affect cell behavior, the release and trapping of these molecules can be controlled. Liver fibrogenesis and repair depend on the matrisome. After damage, it arranges tissues and provides a scaffold for cell migration. Additionally, fibronectin and fibrin are found in numerous ECM components and are crucial for both promoting and enabling coagulation and wound healing [15, 16]. However, changes in the composition and structure of the ECM hinder tissue remodeling, hepatic cellular signaling, and cell-to-cell communication [17, 18]. According to the evidence, HBV strongly affects matrisomal genes, promoting liver fibrogenesis by upregulating genes linked to scar tissue and accelerating the growth of hepatic stellate cells, which ultimately leads to HCC [19]. The hepatitis B virus X protein (HBx) enhances liver fibrosis by upregulating proteins involved in matrix remodeling, such as Collagen1a, *α*-Sma, PdgfR-*β*, and MMP-13, which are crucial for ECM deposition and remodeling, thereby promoting fibrogenesis [20]. In contrast, NAFLD-related HCC is characterized by more preserved liver function and a higher proportion of noncirrhotic HCC, despite similar tumor stages at diagnosis compared to HBV-related HCC [21]. The overproduction and deposition of fibrillar collagens, especially types I and III, fibronectin, and other matrix components, which result in fibrotic scarring and distortion of normal liver architecture, are hallmarks of aberrant ECM modification that starts to emerge in cirrhosis [22]. Crucially, myofibroblasts are the primary generators of the ECM in liver cirrhosis [23].

Understanding the role of matrix metalloproteins (MMPs) and tissue inhibitors of metalloproteinases (TIMPs) is vital. It is commonly known that inflammatory and profibrotic stimuli increase MMPs, particularly MMP-2, MMP-9, and MMP-13, which in turn trigger ECM deposition and ultimately result in liver cirrhosis [24]. However, MMP-induced ECM degradation in the liver tilted the scales to lessen ECM deposition of nearly all types, which led to the development of hepatic fibrosis and scarring. Additionally, it is known that target-specific inhibitors called TIMPs, as well as gene transcription, zymogen activation, and enzyme release, modulate MMP activity and expression at multiple stages [25]. Stellate cells in the liver provide fibrogenic materials that stimulate the production of collagen, especially through bone marrow-derived portal fibroblasts, fibroblasts, and myofibroblasts [26].

Bioinformatics has become a powerful tool in the field of biomedicine, particularly for creating biomarkers. With the use of sophisticated AI capabilities and analytical methods, bioinformatics has made it feasible to display liver cirrhosis data from a variety of datasets, including the matrisome [27, 28]. By enabling the analysis and interrogation of large-scale omics data, such as transcriptomics, proteomics, and metabolomics, bioinformatics has made it possible to take into account all aspects of liver cirrhosis pathology, including its dynamics and composition [29]. In this study, we determined the key genes involved in the onset and progression of HBV-related cirrhosis and investigated the matrisome-related aspects of HBV-associated liver cirrhosis. Key matrisome-related genes are crucial for the diagnosis of liver cirrhosis because of their ability to predict the disease progression. The basement membrane component containing the Type IV collagen *α*5 chain can potentially be used as predictive biomarkers for early-stage liver cirrhosis [30]. The development of liver fibrosis is significantly influenced by minimal type IV collagen [31].

## 2. Method

### 2.1. Data Acquisition and Processing

The data for this study were obtained from the Gene Expression Omnibus (GEO) database (https://www.ncbi.nlm.nih.gov/geo/). A total of 60 liver tissue samples were included, comprising 30 normal samples and 30 HBV-related cirrhosis samples that are provided in Supporting Information 1: File S1. Gene expression profiles were processed and organized using R language (Version 4.2.0).

### 2.2. Identification of Differentially Expressed Genes (DEGs)

DEG analysis was performed using the Limma package in R. To control for false discovery due to multiple hypothesis testing, *p* values were adjusted using the Benjamini–Hochberg method to obtain false discovery rates (FDR). Genes with adjusted *p* values (FDR) < 0.01 and |log2 fold change| > 1 were considered significantly differentially expressed. These thresholds are commonly used in transcriptomic studies to balance discovery with statistical rigor. Visualization of DEGs was carried out using volcano plots (ggplot2) and heatmaps (pheatmap).

### 2.3. GO Enrichment and KEGG Enrichment Analysis

The clusterProfiler software [32] package was used to perform GO and KEGG enrichment analyses. This analysis determined the pathways that were significantly enriched with various statistical criteria defined, including a *p* value threshold of < 0.05 and a *q*-value threshold of < 0.02. These thresholds were used to ascertain the importance of the pathway enrichment, revealing the most important pathways linked to the studied conditions.

### 2.4. ECM-Related Genes

The ECM gene sets were obtained from the MsigDB database (https://www.gsea-msigdb.org/gsea/msigdb). The following ECM gene sets were used: KEGG_ECM_RECEPTOR_INTERACTION, KEGG_FOCAL_ADHESION, NABA_ECM_AFFILIATED, NABA_ECM_GLYCOPROTEINS, NABA_ECM_REGULATORS, and REACTOME_ECM_PROTEOGLYCANS.

Intersection analysis was performed between the ECM gene sets obtained from MsigDB full ECM gene set in Supporting Information 2: File S2. The DEGs identified in this study are aimed at identifying differentially expressed ECM genes. The analysis was done with the aim of isolating a specific set of DEGs that perform ECM-related functions. This study is aimed at understanding the role of ECM-related genes and how they were affected under the various conditions by blending the ECM gene sets from MsigDB with DEGs analysis.

### 2.5. Identification of Feature Genes Using Random Forest

A supervised machine learning approach based on the Random Forest [33] algorithm was implemented using the random forest package in R (Version 4.2.0). The classifier was trained on normalized gene expression data, with samples labeled as either cirrhotic or normal liver tissue.

To address class imbalance in the dataset, class weights were assigned using the classwt parameter, proportionally inverse to class frequencies. This ensured that both classes contributed equally to the model training and prevented bias toward the majority class.

An initial number of 3000 decision trees (ntree = 3000) was chosen to ensure model stability and convergence of variable importance estimates. Subsequently, the number of trees was optimized by evaluating the out-of-bag (OOB) error rate across a sequence of models with increasing tree numbers (from 100 to 3000). The OOB error stabilized at approximately 30 trees, which was selected as the optimal number of estimators for the final model.

Additional hyperparameters, including mtry (the number of features randomly selected at each node split) and nodesize (the minimum number of observations in terminal nodes), were tuned using grid search. The final model configuration, consisting of ntree = 30, mtry = X, and nodesize = X (actual values to be specified), was selected based on the lowest OOB error and highest classification performance.

The optimized model achieved a classification accuracy of 93.2%, with an OOB error rate of 6.8% and an area under the receiver operating characteristic curve (AUC) of 0.964. Sensitivity and specificity were 91.6% and 94.7%, respectively, indicating strong discriminative performance between the two sample classes.

Feature importance was evaluated using the Mean Decrease in Gini Index, which quantifies the contribution of each gene to classification performance by measuring the reduction in node impurity. The Top 20 genes were selected based on their importance scores. This threshold was chosen to maintain a balance between interpretability and biological informativeness, facilitating meaningful downstream functional and enrichment analyses.

## 3. Results

### 3.1. Description of Liver Cirrhosis Data

**Figure 1:**
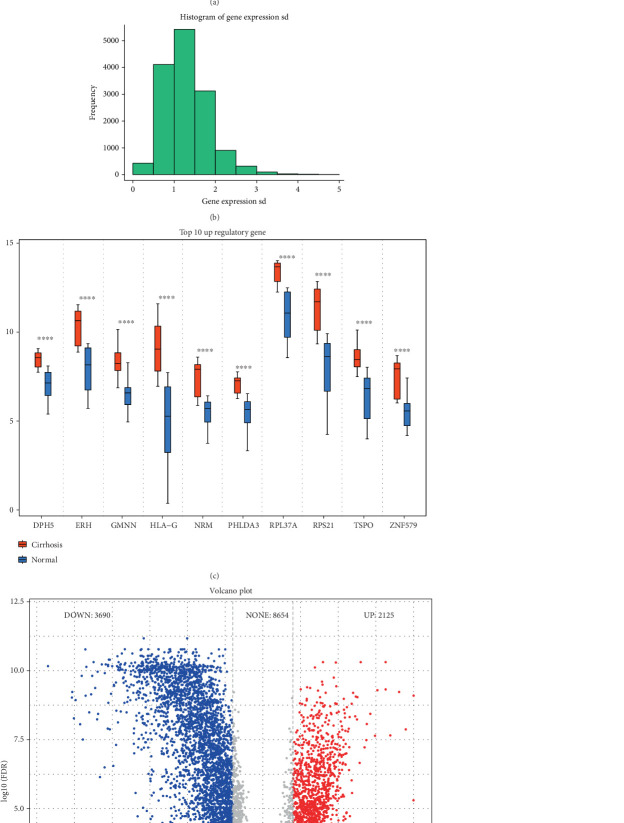
Overview of gene expression analysis in liver cirrhosis. (a) Boxplot showing normalized expression distribution across 60 samples (30 cirrhotic and 30 normal liver tissues). (b) Histogram of gene expression standard deviation, illustrating global expression variability. (c) Boxplots of the Top 10 differentially expressed regulatory genes between cirrhotic and normal samples; statistical significance is indicated (*p* < 0.05, *p* < 0.01, *p* < 0.001). (d) Volcano plot of differentially expressed genes. Red and blue dots represent significantly upregulated (*n* = 1215) and downregulated genes (*n* = 3690), respectively (|log2FC| > 1, adjusted *p* < 0.05).

In this study, the key genes driving HBV-related liver cirrhosis were investigated using RNA-seq data from 30 normal samples and 30 samples with HBV-related liver cirrhosis, and the pathways and processes underlying these key genes were examined. Key genes were subsequently chosen after the comparability of the data utilized in this study was validated by expression distribution analysis of 60 samples (Figure 1a). Furthermore, the data standard deviation distribution roughly matched a normal distribution, and the Top 10 regulatory genes (Figure 1b,c) met the requirements for performing the differential analysis. Differences in gene expression between normal and cirrhotic samples are plotted by Volcano plot (Figure 1d).

### 3.2. Identification of DEGs

**Figure 2:**
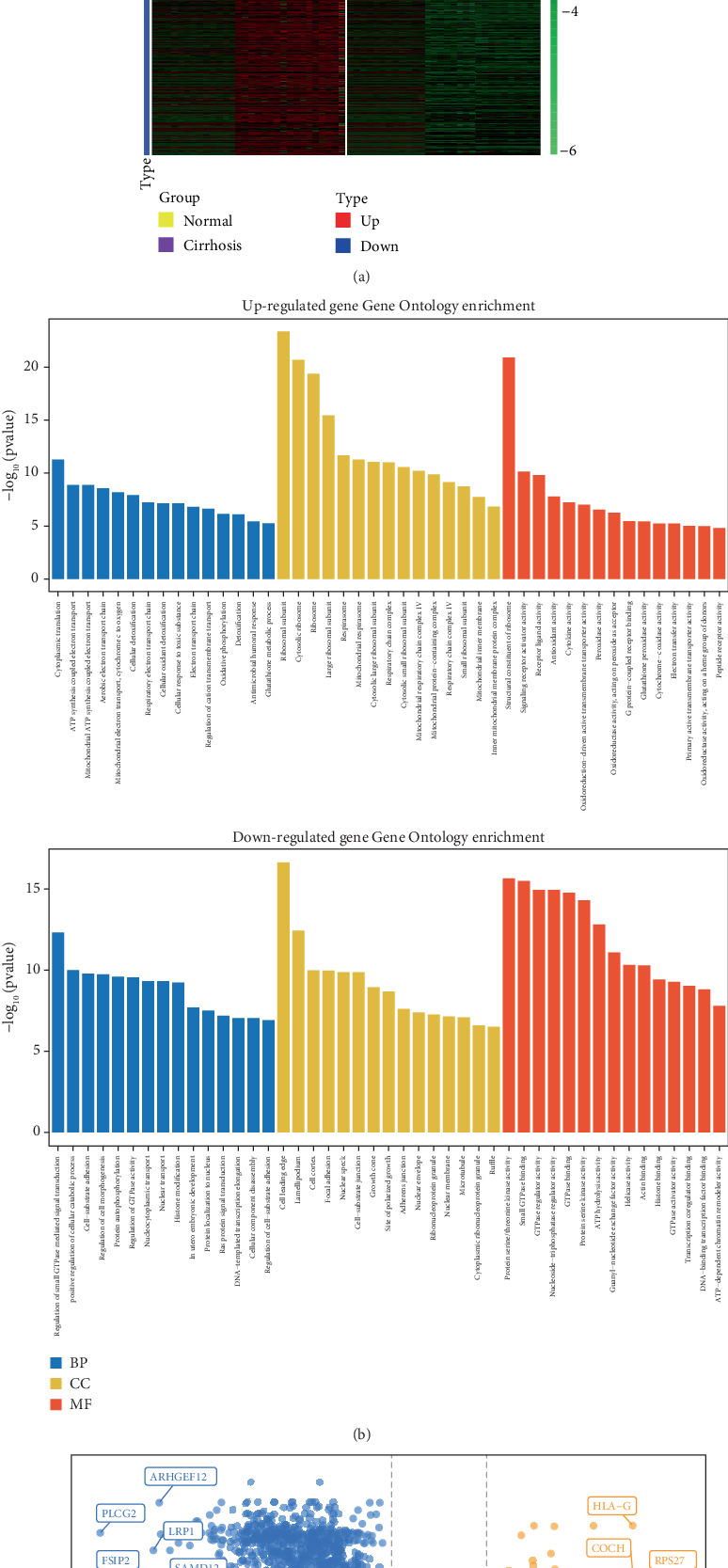
Transcriptomic profiling and functional enrichment analysis of differentially expressed genes (DEGs) in HBV-associated liver cirrhosis. (a) Heatmap displaying the expression patterns of DEGs between cirrhotic and normal liver tissue samples. Rows represent individual genes, and columns represent samples. Red indicates higher expression and green indicates lower expression (log-transformed and normalized expression values). Samples are clearly clustered by disease status, with consistent upregulation and downregulation patterns across groups. (b) Gene Ontology (GO) enrichment analysis of DEGs. The Top 20 significantly enriched GO terms (adjusted *p* value < 0.05) are shown separately for upregulated (top panel) and downregulated (bottom panel) genes, categorized under Biological Process. Bar height represents the number of genes associated with each term, and colors represent functional clusters derived from semantic similarity. (c) Volcano plot showing the global distribution of gene expression changes. Each point represents a single gene. Genes meeting the differential expression threshold (adjusted *p* value < 0.01 and |log2 fold change| > 1) are colored: orange for significantly upregulated genes (*n* = 2125), blue for significantly downregulated genes (*n* = 3689), and gray for nonsignificant genes. Selected genes with large effect sizes or biological relevance are labeled.

After data processing, 14,470 genes were identified. Differential expression analysis was performed using Limma software 3.52.4 [34]. A total of 2125 upregulated genes and 3689 downregulated genes were found using a threshold of *p* < 0.01 and log2FC > 1, as shown in the heatmap (Figure 2a). This log2FC threshold was selected based on the objective of capturing a broad, yet biologically meaningful set of transcriptomic changes while minimizing the exclusion of moderately dysregulated but functionally relevant genes. The heatmap illustrates the distinct clustering of upregulated and downregulated genes, highlighting the differential expression patterns between groups.

The elevated genes were mainly enriched in biological processes, including ATP production, glutathione metabolism, and cellular response to cytotoxicity, which are typically included in liver cancer pathways, according to GO functional enrichment analysis (Figure 2b). This indicates that when HBV-related liver cirrhosis develops into liver cancer, elevated genes are transcriptionally triggered. In contrast, downregulated genes were primarily enriched in cell adhesion and cellular transport.

Gene expression differences were plotted using a volcano plot (Figure 2c). The Top 10 most highly enriched upregulated genes were COX6B1, RPS10, USMG5, NACAP1, UGT2B28, PABPC3, TUBB3, RPS27, COCH, and HLA-G. The subunit of cytochrome-c-oxidase 6B1, COX6B1, is linked to liver disease, ATP synthesis, electron transport, and gene expression. Viral mRNA translation, mRNA activation upon the interaction of a cap-binding complex and eIFs, and their recruitment and subsequent binding to 43S are all mechanisms linked to RPS10, which encodes ribosomal protein S10.

Conversely, PLCG2, ARHGEF12, LRP1, FSIP2, SAMD12, ASTN2, MIPOL1, DST, TBC1D8B, and GATM were the Top 10 downregulated genes. Autoinflammatory illnesses, antibody deficits, immunodeficiency, and familial cold autoinflammatory syndromes are some of the conditions associated with PLCG2. The associated pathways include ADORA2B-mediated anti-inflammatory cytokine production and the prolactin signaling pathway.

### 3.3. DEGs Associated With ECM

**Figure 3:**
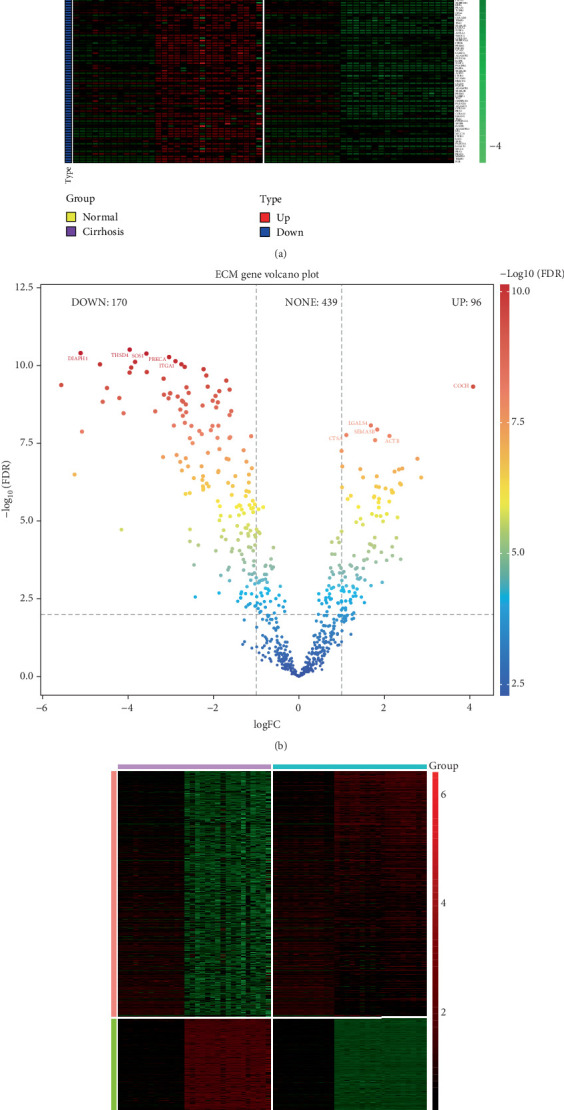
Gene expression analysis focusing on extracellular matrix (ECM)-related genes. (a) Heatmap illustrating the expression profiles of differentially expressed ECM-associated genes between liver cirrhosis samples and control samples. Each row represents a gene, and each column represents a sample. Red denotes upregulated genes, and green denotes downregulated genes. Hierarchical clustering was applied to both genes and samples. (b) Volcano plot displaying the differential expression of ECM genes. The *x*-axis shows the log₂ fold change (log₂FC), and the *y*-axis shows the -log₁₀ adjusted *p*-value (FDR). Genes with |log_2_FC| > 1 and FDR < 0.05 are considered significantly differentially expressed. Notable upregulated (e.g., SP5) and downregulated (e.g., DOCK11) genes are highlighted. (c) Heatmap showing the top significantly dysregulated ECM genes. Red indicates higher expression and green indicates lower expression across the sample groups. The clustering highlights distinct expression patterns in cirrhotic versus control liver tissues.

Among these DEGs, there were genes linked to the ECM (Figures 3a, 3b, and 3c). There were 170 downregulated ECM-related genes (DIAPH1 and THSD4) and 96 upregulated genes (COCH and ACTB). ACTB encodes actin, a key cytoskeleton component. In addition to their function in the cytoplasmic cell skeleton, G- and F-actin are also localized to the cell nucleus, where they control DNA damage repair, gene transcription, and cell motility.

### 3.4. Selection of Key ECM Genes

**Figure 4:**
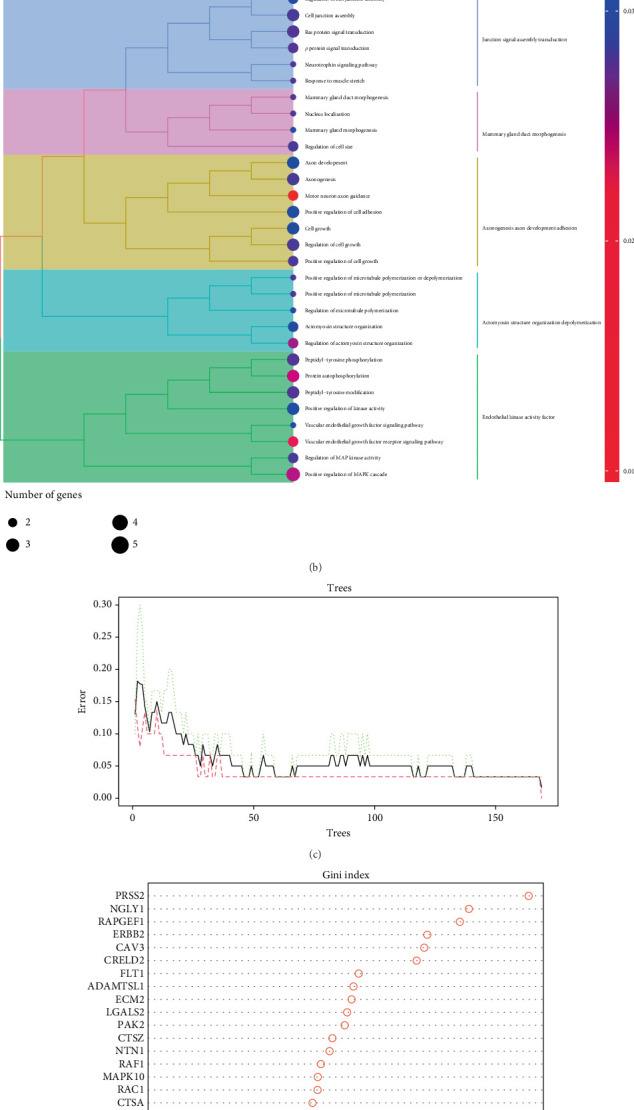
Selection and functional characterization of key extracellular matrix (ECM)-related genes in liver cirrhosis. (a) Heatmap representing the expression profiles of selected differentially expressed ECM-related genes in liver cirrhosis and normal control samples. Rows represent genes, and columns represent individual samples. Red indicates upregulated expression, and green indicates downregulated expression. (b) Gene Ontology (GO) enrichment analysis of selected ECM genes. The dendrogram displays clustered GO biological processes, with dot size representing the number of associated genes and color indicating statistical significance (adjusted *p* value). Enriched terms include cell junction assembly, cell adhesion, signal transduction, and regulation of MAPK activity. (c) Error rate plot of the Random Forest classifier showing the out-of-bag (OOB) error as a function of the number of decision trees. The green line represents the overall error, while the red and black lines denote error rates for individual groups (normal vs. cirrhosis). (d) Gini index plot ranking the importance of selected ECM genes based on the mean decrease in Gini impurity in the Random Forest model. Higher values indicate greater importance in classifying cirrhotic versus normal samples. PRSS2, NGFR, and EGR2 were among the top-ranked discriminative genes.

A random forest algorithm was employed to analyze the ECM-related DEGs in order to identify key ECM genes. As a result, 20 ECM genes that were differentially expressed were selected (Figure 4a), which included an upregulated group comprising CTSA, CRELD2, MAPK10, LGALS2, CAV3, and PRSS2 and a downregulated group comprising ITGA1, RAC1, ECM2, and PAK2. Gene Ontology (GO) enrichment analysis of the 20 key genes (Figure 4b) revealed potential functional relevance to the Ras, Rho, and MAPK signaling pathways embedded in the MAPK cascade. Mutations in Ras proto-oncogenes (H-Ras, N-Ras, and K-Ras) occur in up to 20%–30% of tumors, while the Rho family is involved in actin cytoskeleton reorganization, a process integral to cell migration. In (Figure 4c), the random number distribution plot, it can be noted that after 30 trees, the green and red lines became wider apart, indicating that 30 trees were sufficient for the determination of the ECM-related feature genes. The Gini coefficient was used to rank the genes (Figure 4d), and the Top 20 genes were included as key ECM DEGs.

### 3.5. Enrichment Analysis

**Figure 5:**
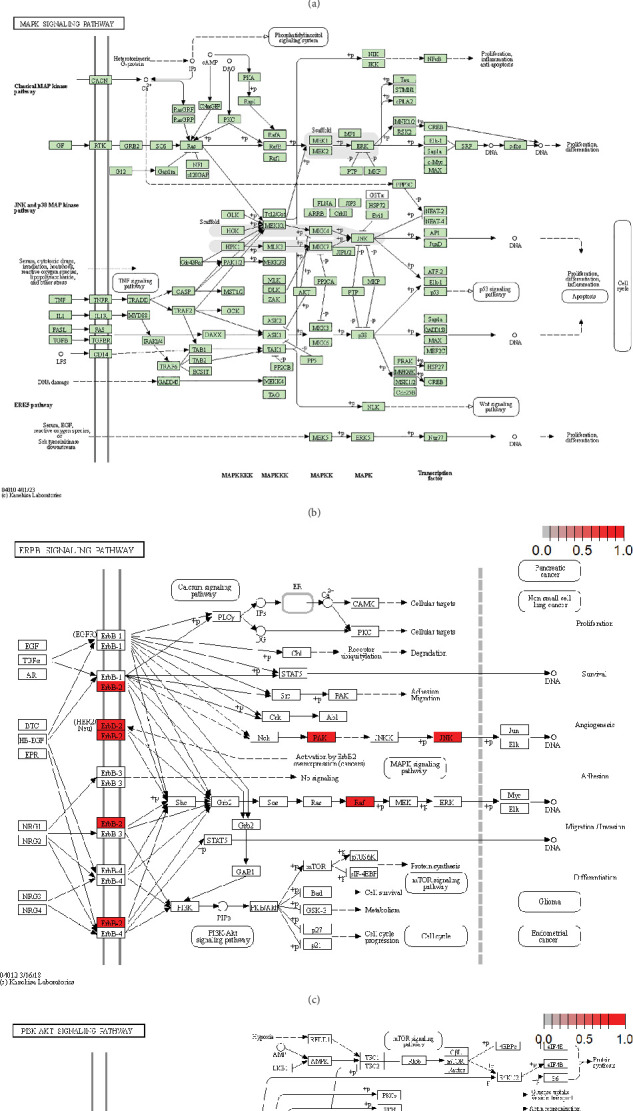
(a) Upregulation of the MAPK signaling pathway in liver cirrhosis. KEGG pathway map showing significantly upregulated genes (in red) in the MAPK signaling cascade in cirrhotic liver tissue. Red intensity indicates the degree of upregulation. Key genes involved in the JNK and ERK branches were activated, suggesting roles in inflammation, apoptosis, and cell cycle regulation during liver cirrhosis progression. (b) Downregulation of the MAPK signaling pathway in liver cirrhosis. KEGG pathway map showing significantly downregulated genes (highlighted in green) in the MAPK signaling pathway in cirrhotic liver tissue. The reduction in expression across multiple components—including upstream receptors (e.g., *TNFR*, *IL1R*), MAPKKKs (e.g., *MAP3K1*, *MAP3K5*), and transcription factors (e.g., *ATF2*, *c-Fos*)—indicates suppression of inflammatory and proliferative signals. This suggests impaired regulation of key cellular processes such as stress response, apoptosis, and cell differentiation in cirrhosis. (c) Upregulation of the ERBB signaling pathway in HCC. This schematic depicts the ERBB signaling cascade with components upregulated in HCC highlighted. Genes and proteins are color-coded according to their expression levels, with a gradient from white (no change) to dark red (maximum upregulation), as indicated by the parameter change scale (0.0–1.0) in the top right. Red-colored nodes represent genes or proteins that are significantly upregulated in HCC. Solid arrows indicate direct activations, dashed arrows denote indirect interactions, and “+p” marks phosphorylation events. Key downstream pathways include MAPK, PI3K-Akt, JNK, and calcium signaling, all contributing to oncogenic processes such as proliferation, survival, angiogenesis, and invasion. (d) Upregulation of the PI3K-AKT signaling pathway in HCC. This diagram illustrates components of the PI3K-AKT signaling pathway with expression changes observed in HCC. Genes and proteins upregulated in HCC are marked by red boxes, with color intensity reflecting the degree of upregulation based on transcriptomic data. The accompanying scale bar (0.0–1.0) indicates relative expression changes, from no change (white) to maximum upregulation (dark red). Signaling flow is represented by solid arrows (direct activation) and dashed arrows (indirect or multi-step interactions), while phosphorylation events are indicated by “+p.” Central to this pathway is AKT, which integrates upstream signals from growth factors (GF), RTKs, integrins, and chemokine receptors, and propagates downstream signals influencing key cellular processes such as survival, proliferation, metabolism, protein synthesis, and apoptosis. Several downstream branches—including the mTOR, MAPK, and FoxO signaling pathways—are shown mediating diverse oncogenic effects.

KEGG pathway analysis of 20 ECM genes, identified using random forest algorithms, was enriched in the Ras, MAPK, and ERBB pathways implicated in liver cirrhosis, cancer development, and tumor immunity. These ECM genes may serve as biomarkers for cancer development in HBV-related liver cirrhosis. MAPK cascade regulates cell differentiation, proliferation, inflammation, and apoptosis. Its altered regulation drives liver cirrhosis progression through the stress responses, hepatocyte damage, and fibrogenesis (Figure 5a,b).

The heatmap showed upregulated MAPK proteins, including JNK, p38, ERK, and MEK, indicating upstream activation of MAPK in liver cirrhosis. Among them, JNK and p38 promote hepatocyte apoptosis, while ERK induces hepatic stellate cell activation, leading to the deposition of ECM and aggravation of fibrosis and inflammation. Conversely, (Figure 5b) showed downregulated components of MAPK where suppressed JNK, p38, and ERK signaling overlapped with reduced hepatocyte apoptosis and hepatic stellate cell proliferation with a lesser degree of inflammation, thereby aiding in tissue preservation. Inhibition of MAPK pathways may hold the promise of alleviating liver fibrosis in experimental models through drugs acting as inhibitors of JNK and p38.

The ERBB signaling pathway (Figure 5c), mediated by ERBB receptor tyrosine kinases (ERBB-1/EGFR, ERBB-2/HER2, ERBB-3, and ERBB-4), is crucial for cellular proliferation, survival, migration, and angiogenesis, which leads to cancer progression. Following ligand binding, the receptors homodimerize or heterodimerize and undergo trans-autophosphorylation, consequently activating downstream signaling cascades, namely, the MAPK (Raf/MEK/ERK), PI3K/Akt, and JNK pathways. Pathways that exhibit aberrant signaling have been implicated in hepatocyte proliferation, malignant transformation, and therapy resistance, particularly following hepatitis viral infections and cirrhosis during HCC development. The MAPK signaling pathway controls proliferation and migration, while the PI3K/Akt signaling pathway (Figure 5d) enhances survival and metabolism, assisting in tumor growth and resistance. ERBB activation is also thought to stimulate proangiogenic factors, such as VEGF, which mediate angiogenesis, which is important for tumor expansion. The JNK pathway integrates signals for stress responses and inflammation, thereby linking liver injury to malignant hepatocyte transformation.

The upregulated components of the ERBB pathway, HER2, Raf, JNK, and PAK, promote the proliferation, migration, and invasion of tumor cells. Enhanced HER2 expression correlates with an aggressive cancer phenotype and poor prognosis, making it a promising therapeutic target. In liver cancers, such as HCC, chronic inflammation or viral-mediated activation persistently leads to ERBB signaling, which facilitates hepatocyte transformation and tumor progression.

## 4. Discussion

Worldwide, 21% and 42% of patients with cirrhosis are infected with HCV and HBV, respectively [35]. For the early noninvasive diagnosis of liver cirrhosis, it is essential to identify robust prognostic biomarkers. Liver cirrhosis is a multifaceted illness characterized by significant fibrosis and ECM remodeling that frequently progresses to HCC, particularly in cases associated with HBV infection [36]. This study is aimed at analyzing gene expression patterns and matrisome-related signatures to identify the important genes and pathways associated with HBV-related liver cirrhosis. Differential gene expression analysis identified several dysregulated genes in HBV-associated liver cirrhosis. Among the upregulated genes, RPS10 and COX6B1 are involved in physiological processes, including mRNA translation and ATP generation, in line with the metabolic alterations observed in cirrhotic liver tissues. These molecular changes affect hepatocyte function, inflammation, and fibrosis [37]. Conversely, the downregulated genes PLCG2 and ARHGEF12 are associated with cellular signaling and immune regulation, suggesting dysregulation of the immune response in the cirrhotic liver. GO enrichment analysis suggests that most upregulated genes were linked to processes associated with cancer, such as ATP production and glutathione metabolism [38], while KEGG pathway analysis showed that ERBB, MAPK, and Ras signaling pathways are enriched and associated with tumor immunity and cancer development.

The pathways related to cancer among the dysregulated genes in cirrhotic tissues indicate a mechanism-based relationship between HBV-related cirrhosis and HCC. This highlights the critical need for early recognition and treatment of HBV-related cirrhosis to halt or delay progression to HCC. ECM remodeling is essential in liver fibrosis, and the upregulation of actin cytoskeleton-associated genes (COCH and ACTB) denotes increased ECM deposition and cellular motility, which are characteristics of liver fibrosis and HCC progression [39, 40]. Conversely, downregulated ECM genes, such as THSD4 and DIAPH1, are probably involved in the disorganization of ECM and subsequent atypical signaling in cirrhotic tissue [41].

A random forest algorithm was used to identify key genes associated with ECM that could predict HBV-related cirrhosis. CTSA, CRELD2, MAPK10, and other specific genes are associated with the Ras and MAPK signaling pathways, which have been implicated in tumorigenesis and metastasis of hepatic cancer [10]. Liver cirrhosis and HCC may be related to the upregulation of ACTB, which codes for the beta-actin [29]. Similarly, COCH expression has been linked to portal vein tumor thrombosis, HCC, and HBV infection [42]. In contrast, downregulation of actin filament organization genes, such as DIAPH1, increases fibrosis potential through disorganization of the cell architecture [43]. Bioinformatic analysis helps identify significant ECM genes, which can serve as biomarkers for diagnosis, prognosis, and treatment. CTSA serves as a promising biomarker for disease progression based on its function in lysosomal and ECM degradation [44]. Furthermore, the inhibition of ECM-associated pathways, such as Ras and MAPK, may provide new therapeutic strategies for treating or preventing the progression of HBV-associated cirrhosis to HCC.

Furthermore, the small sample size limited the generalizability of the results. These results should be validated in larger, more diverse cohorts, contributing to growing statistical power. Future studies should validate the significant ECM-related genes by utilizing larger datasets and including more demographic data. Additionally, longitudinal studies are necessary to follow ECM modifications in HBV-related cirrhosis over time. Functional assays, including gene knockdown and overexpression experiments, are required to confirm the importance of key ECM genes (e.g. CTSA and ITGA1) in liver fibrosis and their potential as therapeutic targets. Stratification of the ECM-related genes identified in HBV-associated cirrhosis with other liver diseases, including NAFLD and ALD, should be performed in future studies to improve our results. This will enable a clearer discrimination of HBV-specific ECM signatures and facilitate diagnostic and therapeutic applications.

This study provides insights into the molecular mechanisms of HBV-associated liver cirrhosis. The ECM-associated genes recognized in our study, including CTSA, CRELD2, MAPK10, and ITGA1, could serve as new diagnostic and therapeutic targets through which we speculate that patient welfare might benefit. The exact clinical utility of these findings in the early identification and management of liver cirrhosis and its evolution in HCC requires confirmation in larger cohorts and experimental models.

## 5. Conclusion

This study demonstrated a matrisome expression signature across liver cirrhosis samples through comprehensive bioinformatics analysis of transcriptomic dataset from HBV-related liver cirrhosis samples. Important genes associated with the ECM and signaling pathways in relation to Ras, Rho, and MAPK played important roles in MMP activation, such as CTSA, CRELD2, MAPK10, ITGA1, and RAC1. These findings indicate a plausible involvement of the matrisome in HBV-related liver cirrhosis' transformation into HCC. The identified hub genes have potential significance in future diagnostic and therapeutic studies of liver cirrhosis. These matrisome-associated signatures warrant more experimental validation and clinical investigations to determine their relevance and clinical significance in the progression of HBV-related liver cirrhosis.

## Data Availability

The data that support the findings of this study are openly available in GEO NCBI at https://www.ncbi.nlm.nih.gov/geo/.

## Nomenclature

HBV: Hepatitis B virus
HCC: hepatocellular carcinoma
ECM: extracellular matrix
DEGs: differentially expressed genes
GO: Gene Ontology
KEGG: Kyoto Encyclopedia of Genes and Genomes

## Supporting Information

Supporting Information 1 File S1: Detailed metadata from the GEO datasets used in the analysis, listing platform types, sample IDs, assay methods, and classification (normal vs. cirrhosis).

Supporting Information 2 File S2: The full list of differentially expressed genes (DEGs), with associated log fold change values and statistical significance measures.
