# Supplementary material for: Identification of Key Gene Related to Matrisome in HBV-Associated Liver Cirrhosis via Bioinformatics Analysis
Source: Int J Hepatol. 2025 Aug 25;2025:5532643. doi: 10.1155/ijh/5532643 (PMC12401599; doi:10.1155/ijh/5532643)
Supplement: Supporting Information 1 — File S1: Detailed metadata from the GEO datasets used in the analysis, listing platform types, sample IDs, assay methods, and classification (normal vs. cirrhosis). [file 5532643.f1.docx]

Table 1 Information of GEO dataset (30 normal samples, 30 cirrhosis samples)

| Platform | Sample | Assay | Type |
| --- | --- | --- | --- |
| GPL6947 | GSM2367554 | Total RNA | Normal |
|  | GSM2367555 | Total RNA | Normal |
|  | GSM2367556 | Total RNA | Normal |
|  | GSM2367557 | Total RNA | Normal |
|  | GSM2367558 | Total RNA | Normal |
|  | GSM2367559 | Total RNA | Normal |
|  | GSM2367560 | Total RNA | Normal |
|  | GSM2367561 | Total RNA | Normal |
|  | GSM2367562 | Total RNA | Normal |
|  | GSM2367563 | Total RNA | Normal |
|  | GSM2367564 | Total RNA | Normal |
|  | GSM2367565 | Total RNA | Normal |
|  | GSM2367566 | Total RNA | Normal |
| GPL24676 | GSM5596632 | Total RNA | Normal |
|  | GSM5596636 | Total RNA | Normal |
|  | GSM5596618 | Total RNA | Normal |
|  | GSM5596620 | Total RNA | Normal |
|  | GSM5596622 | Total RNA | Normal |
|  | GSM5596624 | Total RNA | Normal |
|  | GSM5596626 | Total RNA | Normal |
|  | GSM5596628 | Total RNA | Normal |
|  | GSM5596630 | Total RNA | Normal |
|  | GSM5596634 | Total RNA | Normal |
|  | GSM5596636 | Total RNA | Normal |
|  | GSM5596638 | Total RNA | Normal |
|  | GSM5596640 | Total RNA | Normal |
|  | GSM5596642 | Total RNA | Normal |
|  | GSM5596644 | Total RNA | Normal |
|  | GSM5596646 | Total RNA | Normal |
|  | GSM5596648 | Total RNA | Normal |
| GPL6947 | GSM2367587 | Total RNA | Cirrhosis(Decompensated) |
|  | GSM2367588 | Total RNA | Cirrhosis(Decompensated) |
|  | GSM2367589 | Total RNA | Cirrhosis(Decompensated) |
|  | GSM2367590 | Total RNA | Cirrhosis(Decompensated) |
|  | GSM2367591 | Total RNA | Cirrhosis(Decompensated) |
|  | GSM2367592 | Total RNA | Cirrhosis(Decompensated) |
|  | GSM2367593 | Total RNA | Cirrhosis(Decompensated) |
|  | GSM2367594 | Total RNA | Cirrhosis(Decompensated) |
|  | GSM2367595 | Total RNA | Cirrhosis(Decompensated) |
|  | GSM2367596 | Total RNA | Cirrhosis(Decompensated) |
|  | GSM2367597 | Total RNA | Cirrhosis(Decompensated) |
|  | GSM2367598 | Total RNA | Cirrhosis(Decompensated) |
| GPL570 | GSM437457 | Total RNA | Cirrhosis(Decompensated) |
|  | GSM437460 | Total RNA | Cirrhosis(Decompensated) |
|  | GSM437462 | Total RNA | Cirrhosis(Decompensated) |
|  | GSM437466 | Total RNA | Cirrhosis(Decompensated) |
|  | GSM437473 | Total RNA | Cirrhosis(Decompensated) |
|  | GSM437475 | Total RNA | Cirrhosis(Decompensated) |
|  | GSM437482 | Total RNA | Cirrhosis(Decompensated) |
|  | GSM437492 | Total RNA | Cirrhosis(Decompensated) |
|  | GSM437463 | Total RNA | Cirrhosis(Decompensated) |
|  | GSM437469 | Total RNA | Cirrhosis(Decompensated) |
|  | GSM437471 | Total RNA | Cirrhosis(Decompensated) |
|  | GSM437472 | Total RNA | Cirrhosis(Decompensated) |
|  | GSM437477 | Total RNA | Cirrhosis(Decompensated) |
|  | GSM437479 | Total RNA | Cirrhosis(Decompensated) |
|  | GSM437480 | Total RNA | Cirrhosis(Decompensated) |
|  | GSM437481 | Total RNA | Cirrhosis(Decompensated) |
|  | GSM437484 | Total RNA | Cirrhosis(Decompensated) |
|  | GSM437486 | Total RNA | Cirrhosis(Decompensated) |
